# *Arabidopsis* acyl-acyl carrier protein synthetase AAE15 with medium chain fatty acid specificity is functional in cyanobacteria

**DOI:** 10.1186/s13568-016-0178-z

**Published:** 2016-01-21

**Authors:** Danuta Kaczmarzyk, Elton P. Hudson, Martin Fulda

**Affiliations:** Department of Plant Biochemistry, Albrecht-von-Haller-Institute, Georg-August-University Goettingen, Goettingen, Germany; School of Biotechnology, Science for Life Laboratory, KTH-Royal Institute of Technology, Stockholm, Sweden

**Keywords:** Acyl-ACP synthetase, Medium chain fatty acids, *Arabidopsis*, Cyanobacteria

## Abstract

Cyanobacteria are potential hosts for the biosynthesis of oleochemical compounds. The metabolic precursors for such compounds are fatty acids and their derivatives, which require chemical activation to become substrates in further conversion steps. We characterized the acyl activating enzyme AAE15 of *Arabidopsis* encoded by *At4g14070*, which is a homologue of a cyanobacterial acyl-ACP synthetase (AAS). We expressed AAE15 in insect cells and demonstrated its AAS activity with medium chain fatty acid (C10–C14) substrates in vitro. Furthermore, we used *AAE15* to complement a *Synechocystisaas* deletion mutant and showed that the new strain preferentially incorporates supplied medium chain fatty acids into internal lipid molecules. Based on this data we propose that AAE15 can be utilized in metabolic engineering strategies for cyanobacteria that aim to produce compounds based on medium chain fatty acids.

## Introduction

In recent years metabolic engineering has benefited from advances in gene synthesis and assembly that allow the implementation of complex biosynthetic pathways into a variety of microorganisms (Keasling 2012; Yadav et al. 2012; Seo et al. 2013). One focus of current research is the establishment of biosynthetic pathways for production a variety of oleo compounds such as fatty acids, alcohols, and alkanes in hosts such as yeast, *Escherichia coli*, and cyanobacteria (Steen et al. 2010; Lennen and Pfleger 2013; Pfleger et al. 2015; Savakis and Hellingwerf 2015). A cyanobacteria production host is particularly attractive, as their carbon and energy requirements are minimal. However, cyanobacteria-based production of fatty acids, fatty alcohols and alka(e)nes has been limited to several proof-of-principle studies (Liu et al. 2011; Tan et al. 2011; Ruffing and Jones 2012; Kaiser et al. 2013; Wang et al. 2013; Ruffing 2014; Yao et al. 2014). We strive for the utilization of cyanobacteria for the production of oleochemical compounds. For biosynthetic production of oleochemicals, intrinsically synthesized fatty acids should serve as substrates for the diverse downstream metabolic pathways. In cyanobacteria, fatty acids are chemically activated by acyl-ACP synthetase (AAS) (Kaczmarzyk and Fulda 2010). Acyl-ACP synthetases can therefore play a critical role in metabolic engineering strategies for oleochemicals. In this work we were interested in the closer characterization of an *Arabidopsis* enzyme capable of generating acyl-ACPs and to evaluate its potential for pathway engineering in cyanobacteria.

In *Arabidopsis* enzymes capable of activating fatty acids belong to a superfamily of acyl-activating enzymes (AAEs), which consists of 63 members, and is divided into seven clades based on sequence similarities (Shockey et al. 2003). Clade I contains eleven members and long chain acyl-CoA synthetase (LACS; C16–C20) activity has been confirmed for nine of these (Shockey et al. 2002). The conversion of very similar fatty acid substrates is reflected by characteristic features of the amino acid sequences of the proteins of clade I. In particular, clade I AAEs differ from all other AAEs by the presence of an amino acid stretch separating two highly conserved sequence motifs. Interestingly, this amino acid linker is remarkably longer in the two remaining proteins of clade I, for which initial tests were unable to proof LACS activity (Shockey et al. 2002). These proteins called AAE15 and AAE16 and encoded by *At4g14070* and *At3g23790*, respectively, include an amino acid linker of approximately 70 amino acid residues, compared to about 40 amino acids found in eukaryotic LACSs (Shockey et al. 2002).

It was proposed previously that *Arabidopsis* AAE15 is a plastidial AAS (Koo et al. 2005). The conclusions were drawn from experiments in which plant extracts of *Arabidopsis* wild type and *AAE15* and *AAE16* knock-out lines were incubated in the presence of radioactive labeled medium chain fatty acids. We showed later that acyl activating enzymes characterized by the presence of a linker motif of 68–74 amino acid residues indeed have AAS activity (Kaczmarzyk and Fulda 2010). Sequences of this type could be found in sequenced genomes of almost all organisms performing oxygenic photosynthesis.

In a recent report, Beld et al. (2014) analyzed the activity of *Arabidopsis* AAE15 using a more direct approach. The enzyme was expressed in *E. coli*, and tested in acyl-CoA synthetase and AAS assays. It was concluded that *At*AAE15 was a poor enzyme in both assays (Beld et al. 2014).

We were interested in further characterization of *Arabidopsis* AAE15, and its activity in *Synechocystis* sp. PCC6803. In this work, we expressed AAE15 heterologously in insect cells, purified it, and analyzed its enzymatic activity in vitro. We demonstrated AAS activity for AAE15 with some specificity for medium chain fatty acids (C10:0–C14:0). Moreover, we expressed AAE15 in the background of an AAS deletion mutant of *Synechocystis* sp. PCC6803. This mutant is unable to incorporate exogenously added fatty acids into lipids, and secrete free fatty acids to the culture media (Kaczmarzyk and Fulda 2010). Feeding experiments with radiolabeled fatty acids confirmed medium chain fatty acid specificity of AAE15.

## Materials and methods

### Heterologous expression of tagged AAE15 in insect cells

For heterologous expression the Bac to Bac Baculovirus Expression System (Thermo Fisher Scientific) was used. Two variants of *AAE15 (At4g14070)* were cloned in frame with the N-terminal 6xHis tag of the pFastBac™HT. The first clone corresponds to the complete open reading frame including the native start codon. For the second clone the predicted plastidial targeting signal was removed, leading to an N-terminal deletion of 195 bps. The vector pUNI51 carrying *At4g14070* served as a PCR template, and full length and truncated versions of the gene were amplified using a forward primer introducing a *Nco*I restriction site, and a reverse primer including the stop codon, introducing a *Not*I restriction site. The primers sequences were 5′-AGATCCATGGAAATTCGTCTGAAACCT-3′ (forward 1), 5′-AGTACCATGGCTTGCGAGTCAAAGGAAAAAGAAG-3′ (forward 2), and 5′-AGTAGCGGCCGCTTAACTGTAGAGTTGATCAATC-3′ (reverse). PCR products were cloned into pGEMT-vector (Promega), verified by sequencing, and subsequently transferred into pFastBac™HT. The vectors were used to transform competent DH10Bac *E. coli* cells. Bacmid DNA was isolated and used to transfect Sf9 cells. A recombinant Baculovirus stock P1 was used to infect cells to produce a P2 Baculovirus stock, which was titered and used to infect insect cells for protein expression. Sf9 cells were infected at MOI 3 and grown at 27 °C as adherent cultures in T-75 culture flasks using Sf-900 II SFM media supplemented with penicillin at 50 U mL^−1^, and streptomycin at 50 μg mL^−1^.

### Isolation and purification of recombinant protein from insect cells

Cells from two T-75 flasks were harvested 72 h after infection, washed once with PBS, and resuspended in 1 mL of extraction buffer (50 mM Tris–HCl pH 7.8, 150 mM NaCl). Cells were disrupted by sonication (2 × 30 s on ice) with Branson Sonifier Cell Disruptor B15, and cell debris was removed by centrifugation at 3500*g* at 4 °C for 15 min. Aliquots of the supernatant were saved for Western blot analysis and activity assays, and the remaining volume was centrifuged at 100,000*g* at 4 °C for 1 h to isolate the membrane fraction. The membranes pellet was resuspended in 300 μL of solubilization buffer (50 mM Tris–HCl, pH 7.8, 150 mM NaCl, 2 % Triton X-100), incubated at 4 °C overnight with agitation to release membrane-bound proteins, and clarified by centrifugation at 100,000*g* at 4 °C for 30 min. To purify His-tagged proteins the supernatant was applied to 800 μL of BD TALON resin (BD Biosciences) and agitated for 4 h at 4 °C to enable protein binding. The resin was transferred to a gravity-flow column and washed first with the solubilization buffer, and then with the same buffer supplemented with 20 mM imidazole to remove non-specifically bound proteins. The target protein was eluted with the solubilization buffer containing 100 mM EDTA. Fractions of 200 μL were collected and dialyzed overnight against 400 mL of the solubilization buffer at 4 °C. Protein concentration in cellular lysates and membrane suspensions was determined using Bradford assays. Protein concentration in the sample of the purified protein was not determined.

### Immunoblot analysis

Protein samples were separated on standard 10 % SDS polyacrylamide gels and transferred to the Optiran BA-S 83 membrane (Schleicher and Schuell). Membranes were blocked with 3 % BSA in TBST buffer (10 mM Tris–HCl, 150 mM NaCl, 0.1 % Tween 20, pH 8.0), and probed with TetraHis-Antibody (Qiagen). As secondary antibody a peroxidase conjugated anti-mouse antibody was employed and the signals were detected by chemiluminescence using ECL Western Blotting Kit (Amersham).

### Enzyme assays

The AAS activity was measured according to the protocol described before (Rock and Cronan 1981). The assay buffer contained 2.5 mM Tris–HCl (pH 8.0), 2 mM dithiothreitol, 0.25 mM MgCl_2_, 5 mM ATP, 10 mM LiCl, 2 % Triton X-100, 15 μM acyl-carrier-protein (ACP; from *E. coli* K12), and 30 μM [1-^14^C] fatty acid (specific activity 53.7–60 mCi mmol^−1^) in a total volume of 40 μL. The assays were initiated by adding defined amounts of protein sample (50 μg of total protein when crude cellular extracts were used as source of enzyme, and 10 μL of purified protein), and were conducted at 37 °C for 30 min. Transferring the assay volume to filter disks stopped the assays. The filter disks were dried and subsequently washed twice with 20 mL of chloroform: methanol: acetic acid (3:6:1, v/v/v) to remove unreacted free fatty acids. Control assays using only free fatty acids demonstrated quantitative removal of the labelled fatty acids by the two washing steps. The radioactivity was determined by liquid scintillation counting (Liquid Scintillation Analyser 1900 TR, Fa. Canberra Packard).

To make sure that all fatty acid substrates are accessible to the enzyme, positive control assays were performed, in which purified AAS from *Synechococcus elongatus* PCC 7942 was used. *Synechococcus* AAS was characterized before, and showed broad substrate specificity (C12–C18) (Kaczmarzyk and Fulda 2010).

### Lipid analytical methods

Pre-cultures of *Synechocystis* wild type and mutant strains were diluted to OD_730_ 0.2 in 15 mL BG11, and cultures were grown for 3 days. Cells of 10 mL culture were harvested, and washed twice in 0.1 M NaHCO_3_. Intracellular and extracellular lipid extractions were performed according to the protocol established before (Bligh and Dyer 1959). Fatty acids were converted to their methyl esters according to modified protocols described earlier (Christie 1982; Stumpe et al. 2001). The fatty acid methyl esters were analyzed by gas chromatography using a Shimadzu GC-2010 gas chromatograph equipped with a Stabilwax column (Restek).

### Fatty acid uptake assay

Cyanobacterial cells were collected from 10 mL cultures at OD_750_ 1 by centrifugation, resuspended in 2 mL of fresh BG11 medium, and transferred to a 2 mL microcentrifuge tubes. Radiolabeled [1-^14^C] fatty acids (lauric, specific activity 57 mCi mmol^−1^, myristic 55 mCi mmol^−1^, palmitic 60 mCi mmol^−1^, stearic 58 mCi mmol^−1^, oleic 56 mCi mmol^−1^, linolenic 53.7 mCi mmol^−1^; Amersham Biosciences) were individually added in amounts corresponding to 0.22 μCi, and the tubes were placed on a platform shaker under light and incubated for 15 h. Cells were pelleted and washed twice with 0.1 M NaHCO_3_. Total lipid extracts were prepared as follows: 1.5 mL chloroform: methanol (2:1, v/v) acidified with HCl were added to the cell pellets in 2 mL tubes, and lipids were extracted for 4 h under shaking. Afterwards 500 μL 0.45 % NaCl was added, the tubes were shaken briefly, and centrifuged at 2000*g* for 2 min for phase separation. The lower phase was transferred to a new tube, dried under a stream of nitrogen and resuspended in 20 μL of chloroform: methanol (1:1, v/v). Different lipid classes were separated by thin layer chromatography using acetone: toluene: water (91:30:8, v/v/v) as solvent and were visualized by fluorography. Signals were detected with an image analyzer (FLA-3000, Fujifilm).

### Cyanobacteria strains and growth conditions

Liquid cultures of the glucose-tolerant *Synechocystis* sp. PCC 6803 and mutant strains were grown photoautotrophically in BG11 media buffered to pH 7.8 with 25 mM HEPES at 30 °C, with 45 μE s^−1^ m^2^ illumination in a climatic chamber (Percival Climatics SE-1100). For fatty acid profiles analysis, cultures were grown under 1 % (v/v) CO_2_ conditions. Mutant strains were cultivated in BG11 containing an appropriate antibiotic for the selection (kanamycin 25 μg mL^−1^, and/or chloramphenicol 20 μg mL^−1^). To prepare solid media 0.3 % (w/v) sodium thiosulfate pentahydrate and 1.5 % (w/v) agar were added to the buffered BG11 media. The plates were incubated under illumination with 25 μE s^−1^ m^2^.

A ∆*aas* deletion strain, in which a kanamycin resistance cartridge replaced part of the coding region of the gene *slr1609*, was created before (Kaczmarzyk and Fulda 2010). This strain was used as a host to overexpress homologous acyl activating enzyme from *Arabidopsis thaliana*: AAE15 (*At4g14070*).

In the first strategy the *Arabidopsis* gene was introduced into the cyanobacterial genome via homologous recombination. To this end, expression constructs were prepared which contained a promoter of a kanamycin resistance gene, the *ArabidopsisAAE15* gene, the terminator sequence of a native *aas*, and the chloramphenicol resistance gene as a selection marker. The whole assembly was flanked by fragments of the kanamycin resistance gene, which served as homology regions for the recombination. The list of primers used for amplifying those blocks is provided in Table 1. The kanamycin resistant ∆*aas* strain was transformed with a pUC19 vector carrying the expression construct, and after few rounds of re-streaking on BG11 agar plates containing chloramphenicol, fully segregated complementation strain ∆*aas*:*AAE15* was obtained. The complete segregation was confirmed by PCR.

**Table 1:** List of primers

Name	Sequence
KanF1Sac	ACGAGAGCTCGCCCCATCATCCAGCCAGA
KanR1Xba	CTGAATCTAGATATTCTTCTAATACCTGG
KanF2Hind	CTAGTAAGCTT GCGCCGGTTGCATTCGA
KanR2Kpn	TGAATGGTACC ATCATCCAGCCAGAAAGT
KanProSpeF	GACTAGTGAATCGCCCCATCATCCAGCCA
KanProNcoR	TCCATGGCACCCCTTGTATTACTGTTTATG
AAE15F	AGATCCATGGAAATTCGTCTGAAACCT
AAE15R	AGTAGCGGCCGCTTAACTGTAGAGTTGATCAATC
SYN68TermFEcoNot	GTAGAATTCGCGGCCGCTTAAGAACCTGTTTATAAAGTCT
SYN68TermRHind	GAACTTGCCGCAAGCTTACTC
JOANRT15	TTTCCCTGGTGATTTCTTCG
JOANRT16	ATATGCCCTGGGAGGGTTAC
DAKART01	CGATGGCTTGTTTCAGATCA
DAKART02	ATGCGGTTGAAAAACTCAGG
DAKART03	GCCACCCTGATCTACACCTC
DAKART04	TTCTAGGGAGTGCCAACAGG

In the second strategy the ∆*aas Synechocystis* host was transformed with a replicative plasmid pJA2c, carrying *AAE15*, devoid of the plastidial targeting signal, under the control of *psbA2* promoter. The pJA2c vector was constructed by Huang et al. (2010), and modified later (Anfelt et al. 2013), and contains chloramphenicol resistance gene as a selection marker. The primers used for amplification of *AAE15* were as follows: forward (adding *Xba*I restriction site) 5′-GACCTCTAGAATGTGCGAGTCAAAGGAAAAAGAAG-3′, reverse (adding *Spe*I restriction site) 5′-CTACACTAGTTTAACTGTAGAGTTGATCAATC-3′.

### RT-qPCR

*Synechocystis* cells were collected from 6 mL cultures at OD_730_ 1, and total RNA was isolated with GeneJET RNA Purification Kit (Thermo Scientific) according to manufacturer’s instructions with the following modifications: lysozyme concentration in TE buffer was 40 mg L^−1^, and cells were disrupted by vortexing with glass beads for 15 min. DNA was removed with RapidOut DNA Removal Kit (Thermo Scientific).

RT-qPCR was performed in the CFX96 Real-Time PCR Detection System (Bio-Rad) with iScript One-Step RT-PCR Kit with SYBR Green (Bio-Rad). All reactions were performed in duplicate, and no-RT controls were included. As a reference gene *rpoB* (*sll1787*) encoding RNA polymerase beta subunit was used. The list of primers is provided in Table 1.

## Results

### AAE15 has AAS activity in vitro, with specificity for medium chain fatty acids

**Fig. 1:**
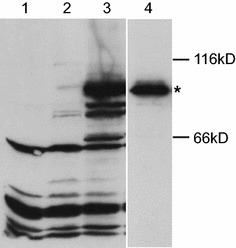
Western blot analysis of *Arabidopsis* AAE15 expressed in insect cells. His-tagged AAE15 devoid of plastidial targeting signal was expressed in insect cells. Samples of whole cell lysate and purified protein were subjected to immunoblot analysis employing anti-His antibody. The *lanes* represent crude protein extracts of uninfected cells (*1*) and cells infected with the empty vector (*2*) as controls and extracts of cells expressing AAE15 (*3*), and the purified AAE15 protein (*4*). The sizes of molecular mass standards are indicated to the *right*. AAE15 protein is marked by *asterisk*

To determine the in vitro enzymatic activity of AAE15, the protein fused to an N-terminal polyhistidine-tag was expressed in a Baculovirus system. Attempts to express the complete open reading frame in Sf9 cells resulted in either very low or undetectable amounts of recombinant protein. Removing the sequence encoding a predicted plastidial targeting peptide increased the expression level significantly, and the construct yielded fusion protein of the expected size ~79 kD (Fig. 1).

**Fig. 2:**
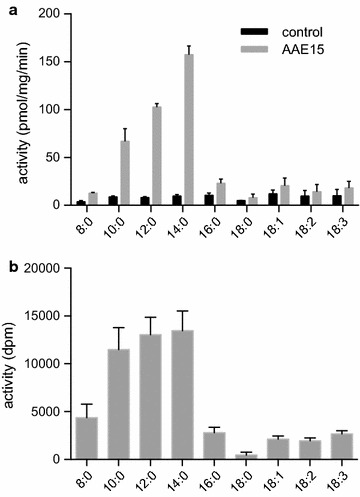
Acyl-ACP synthetase activity assays of AAE15 expressed in insect cells. Crude extracts of Sf9 cells (**a**), and purified AAE15 (**b**) were incubated with an assay mixture containing ACP, ATP and [1-^14^C] fatty acids for 30 min, at 37 °C. As control cells infected with the empty vector were used. AAE15 lacks the predicted plastidial targeting signal. Each measurement was performed in triplicate. *Error bars* indicate standard deviation

The crude protein extract of lysed cells expressing AAE15 was analyzed in enzymatic activity assays. The assays were conducted in the presence of ATP, ACP and [1-^14^C] labeled fatty acids. We tested nine linear fatty acids, ranging in chain length from 8 to 18 carbon atoms and containing between 0 to 3 double bonds. AAE15 demonstrated AAS activity with medium chain fatty acids (Fig. 2a). Specific enzyme activities were 66.7 (SD 13.3), 102.7 (SD 3.6), and 157.2 (SD 9.2) pmol min^−1^ mg^−1^ total protein for decanoic, lauric and myristic acid substrates, respectively. This substrate specificity assay was repeated with *purified* AAE15 (Fig. 2b), and the result confirmed the specificity for medium chain length fatty acids. Moreover, the data proved that AAE15 was able to activate other fatty acids as well, but with clearly reduced efficiency.

### Complementation of *Synechocystisaas* knockout with *ArabidopsisAAE15*

In order to examine the activity of *Arabidopsis* AAE15 in vivo, we performed feeding of radioactive fatty acids. A *Synechocystis* strain lacking its endogenous AAS (∆*aas*) was used as a host to express *ArabidopsisAAE15*. The *AAE15* expression cassette was integrated into the chromosome of the cyanobacteria ∆*aas* strain by homologous recombination to create ∆*aas*:*AAE15.* Complete segregation of the newly generated strain was confirmed by PCR. Wild type *Synechocystis* and the ∆*aas* strain served as positive and negative control, respectively in fatty acid uptake assays.

**Fig. 3:**
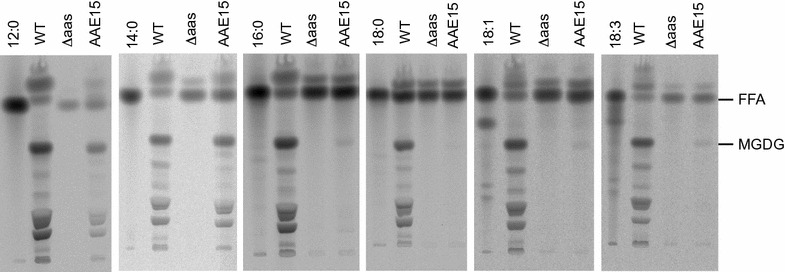
Autoradiography of total lipid extracts from *Synechocystis* ∆*aas:AAE15*. Cultures of *Synechocystis* sp. PCC 6803 wild type (WT), *aas* knock-out strain (∆*aas*), and *aas* knock-out strain complemented with *Arabidopsis* *AAE15* (AAE15) were grown in presence of [1-^14^C] labeled fatty acids. Lipid extracts of harvested cells were separated by TLC. *FFA* free fatty acid, *MGDG* monogalactosyldiacylglycerol, (*C12:0* lauric acid, *C14:0* myristic acid, *C16:0* palmitic acid, *C18:0* stearic acid, *C18:1* oleic acid, *C18:3* linolenic acid). The *spot* above the free fatty acids was proven to be fatty acid methyl ester and originated from the extraction method applied

Cultures were grown in media supplemented with radiolabeled fatty acids, and subsequently total lipid extracts of the cells were separated by thin layer chromatography in order to trace the fate of the supplemented fatty acids. All tested fatty acids were incorporated into different lipid classes in wild type cells, while in the ∆*aas* mutant strain the label strictly remained in the fraction of free fatty acids. In absence of the endogenous AAS protein, the supplied free fatty acids were absolutely inaccessible to the cellular metabolism. The expression of AAE15 in the ∆*aas* mutant strain could partially restore the wild type phenotype indicated by the incorporation of fatty acids into lipids (Fig. 3). The intensities of the spots corresponding to monogalactosyl diacylglycerol (MGDG), the main lipid fraction in extracts, of the complemented strains were clearly weaker compared to those of wild type extracts, but they proved the capability of AAE15 to activate exogenously added free fatty acids (Table 2). The endogenous AAS of *Synechocystis* mediated the incorporation of the different chain-length radiolabeled fatty acids with comparable efficiency, while ∆*aas:AAE15* preferentially incorporated lauric acid and myristic acid. Thus, the in vivo experiments confirmed the results of the in vitro activity assays showing preference of AAE15 for medium chain fatty acids and demonstrated AAE15 activity in *Synechocystis*.

**Table 2:** Intensities of spots representing FFA and MGDG

	WT		∆*aas*		∆*aas:AAE15*	
	FFA	MGDG	FFA	MGDG	FFA	MGDG
12:0	137.4	989.7	36.6	0.3	49.7	57.5
14:0	82.1	252.2	273.3	0	260.9	139.6
16:0	372.4	968.3	2689	3.2	4171.5	10.1
18:0	1665.7	205.7	919.3	1.2	1090.7	2.3
18:1	88.4	294.8	642.9	1.1	687.1	2.9
18:3	32.6	223.8	63.3	0.8	61.2	3.1

Radiolabeled fatty acids were fed to cells of *Synechocystis* sp. PCC6803 wild type (WT), the *aas* knockout mutant (∆*aas*), and the *aas* mutant complemented with *Arabidopsis AAE15* (∆*aas:AAE15*)

### Expression of *ArabidopsisAAE15*, resulted in changes in intracellular and extracellular free fatty acids pools in the cyanobacterial ∆*aas* strain

**Fig. 4:**
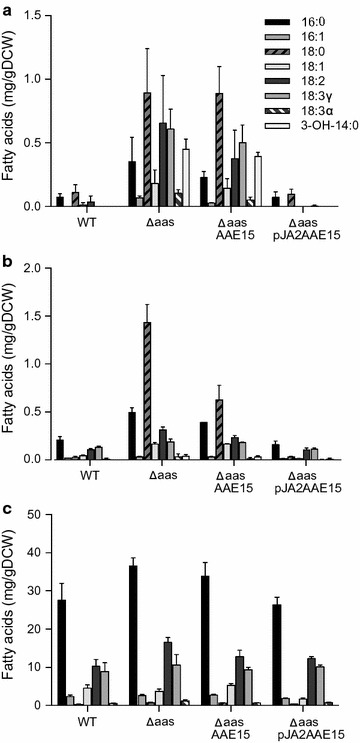
Fatty acid profiles of cells and culture media of the different *Synechocystis* strains. **a** Free fatty acids in the culture media. **b** Free fatty acids within the cells. **c** Esterified fatty acids of the cellular lipid extract. *Synechocystis* sp. PCC 6803 wild type (WT); *aas* knockout strain (∆*aas*); and *aas* knock-out strain complemented with *AAE15* from *Arabidopsis* (∆aas:AAE15, complete *AAE15* integrated into the genome; ∆aas:pJA2AAE15, replicative plasmid encoding the truncated *AAE15* under control of the psbA2 promoter)

The fatty acid uptake assays demonstrated the ability of *Arabidopsis* AAE15 to activate exogenously added fatty acids of different carbon chain length. We next evaluated how the expression of AAE15 influenced the fatty acid metabolism in cyanobacteria. We analyzed the *secreted* fatty acids of ∆*aas*:*AAE15* and compared this to ∆*aas*, and wild type *Synechocystis*. The results showed that like ∆*aas*, ∆*aas*:*AAE15* secreted large amounts of fatty acids into the culture medium (Fig. 4a). Total free fatty acids after 3 days were 3.3 (SD 1.0) mg g^−1^ of dry cell weight (DCW) for ∆*aas*, and 2.6 (SD 0.7) mg g^−1^ DCW for ∆*aas*:*AAE15* (Fig. 4a). The *intracellular* pool of free fatty acids of ∆*aas*:*AAE15* was also similar to ∆*aas*, namely an increased amounts of total free fatty acids and a significant accumulation of 18:0 in comparison to the wild type was observed (Fig. 4b). Concentrations of *internal* free fatty acids were 2.7 (SD 0.3) mg g^−1^ DCW for ∆*aas*, 1.7 (SD 0.2) mg g^−1^ DCW for ∆*aas*:*AAE15,* and 0.5 (SD 0.1) mg g^−1^ DCW for wild type. Interestingly, free stearic acid (C18:0) was 50 % lower in the ∆*aas*:*AAE15* strain, compared to ∆*aas* (Fig. 4b). Overall, expression of *AAE15* in the ∆*aas* strain did not affect fatty acid profiles and did not have a significant complementation effect.

**Fig. 5:**
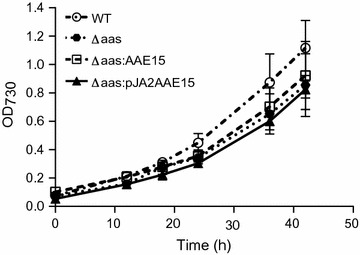
Growth curves of wild type and mutant strains. Cultures were grown in triplicate under standard conditions and OD_730_ was monitored at six time points; *Synechocystis* sp. PCC 6803 wild type (WT); *aas* knockout strain (∆aas); and *aas* knock-out strain complemented with *AAE15* from *Arabidopsis* (∆aas:AAE15, genome integration; ∆aas:pJA2AAE15, replicative plasmid)

Since ∆*aas*:*AAE15* did not show significant differences to ∆*aas* in vivo, we attempted to increase *AAE15* expression levels by using a replicative plasmid, and a stronger promoter. Additionally, we removed the plastidial targeting sequence, to express exactly the version of the protein as it is found in plastids of *Arabidopsis* (Zybailov et al. 2008). Before, we observed that removing the transit peptide resulted in more robust expression levels in insect cells. The truncated *AAE15* was then cloned into an episomal vector (pJA2c) under control of the strong promoter P*psbA2*. This plasmid was transformed into ∆*aas* to give ∆*aas*:pJA2*AAE15.* We analyzed the expression level of *AAE15* in mutant strains, and found that *AAE15* expression was 170 fold higher in ∆*aas*:pJA2*AAE15* compared to ∆*aas*:*AAE15* (Table 3). The increased expression resulted in the complete reversion of the biochemical phenotypes of the ∆*aas* strain when complemented with *AAE15* (Fig. 4). The strain ∆*aas*:pJA2*AAE15* did not secrete free fatty acids and the profile of the intracellular free fatty acids was similar to wild type, with very low concentrations of free stearic acid. The concentration of intracellular fatty acids 0.4 (SD 0.1) mg g^−1^ DCW was also similar to wild type cells (Fig. 4a, b). These results suggest that expression of AAE15 in ∆*aas*:pJA2*AAE15* was high enough to overcome its poor preference for long chain fatty acids. The profile of membrane bound fatty acids in wild type and mutant *Synechocystis* strains did not show any significant differences (Fig. 4c). Growth rates of all strains investigated did not show any significant differences either (Fig. 5).

**Table 3:** Gene expression level for acyl activating enzymes in different strains

Strain	Gene	Abundance (gene/rpoB)
WT	*aas*	0.30
∆*aas:AAE15*	*AAE15*	0.09
∆*aas:pJA2AAE15*	*AAE15*	14.84

## Discussion

In this work we characterized the *Arabidopsis* AAE15 enzyme in *Synechocystis* sp. PCC6803. We were particularly interested in evaluating the possibility to introduce modified substrate specificity into the cyanobacterial fatty acid metabolism. To obtain first insight into its enzymatic parameters we expressed *Arabidopsis* AAE15 in insect cells, and determined its AAS activity in vitro. In a recent report it was concluded upon heterologous expression in *E. coli* that the enzyme possesses poor activity in both acyl-CoA synthetase and AAS assays (Beld et al. 2014). In that study the full-length protein was expressed in the *E. coli* strain BL21. We propose that removing of the N-terminal transit peptide is essential to detect a robust AAS activity of AAE15 in expression hosts other than plants. A construct expressing such truncated protein resulted in significant AAS activity with decanoic, lauric and myristic acid (Fig. 2a, b).

It was hypothesized previously that AAE15 may be involved in acyl editing of membrane lipids in *Arabidopsis* cells (Koo et al. 2005). Thus, in addition to medium chain fatty acids, we tested fatty acid substrates typically found in *Arabidopsis* lipids: C16, and C18 fatty acids with 0–3 double bonds. Our in vitro activity assays showed that AAE15 is an AAS with strong preference for C10, C12, and C14 substrates, but can also activate other fatty acids with greatly reduced efficiency. The results indicated considerably different substrate specificity compared to the *Synechocystis* endogenous AAS (Kaczmarzyk and Fulda, 2010).

The in vitro data was confirmed by in vivo experiments. When we replaced the native *aas* gene of *Synechocystis* by *AAE15* and fed the mutant strain with labeled fatty acids the results again indicated a very clear preference for medium chain fatty acids. In contrast, the endogenous AAS activity of the *Synechocystis* wild type strain mediated comparable incorporation of all offered fatty acids and showed no particular substrate specificity (Fig. 3).

On the other hand, our data showed also that a more robust expression of AAE15 is able to complement the inactivation of the endogenous AAS protein in *Synechocystis*. When the truncated version of AAE15 lacking the plastidial targeting signal was expressed under control of the strong *psbA2* promoter the fatty acid secretion phenotype of the cyanobacterial *aas* knockout strain was revoked, indicating that all fatty acids that could be detected in the culture media of the ∆*aas* strain, were activated and recycled in the strain complemented, ∆*aas*:pJA2*AAE15*.

The AAE15 enzyme could be a useful tool for metabolic engineering projects aimed at the biosynthesis of medium chain fatty acid-derived products. There has been growing interest in engineering microorganisms for fatty acid-derived chemicals and fuels (Steen et al. 2010; Lennen and Pfleger 2013; Pfleger et al. 2015; Savakis and Hellingwerf 2015). One of the challenges is to tailor the carbon chain length in order to obtain the desired properties of the final fatty acid-derived products. For example, medium chain length fatty acids are extensively used for the production of soap and detergents (Dyer et al. 2008), and medium chain length alkanes are main components of jet fuel (Kallio et al. 2014). Reports addressing the chain length issue propose expression of acyl-ACP thioesterases with medium chain fatty acids specificity, as enzymes that can control the length of the end product (Zheng et al. 2012; Choi and Lee 2013; Howard et al. 2013; Liu et al. 2013; Torella et al. 2013; Youngquist et al. 2013) Enzymes involved in oleochemical biosynthesis pathways usually require a CoA- or ACP-activated derivative of the fatty acid substrate. In cyanobacteria, fatty acid metabolism relies on ACP-thioesters, which are the preferred substrates of acyl transferases (Weier et al. 2005) in lipid synthesis, and the acyl-ACP reductase of the alkane synthesis pathway (Schirmer et al. 2010). A strategy aimed at the production of medium chain length fatty alcohols in *E. coli* was published recently (Youngquist et al. 2013). An AAS such as AAE15 that can efficiently deliver activated medium chain fatty acids to downstream metabolic pathways is of significant biotechnological interest.
